# The efficacy of DNA mismatch repair enzyme immunohistochemistry as a screening test for hypermutated gliomas

**DOI:** 10.1186/s40478-020-0892-2

**Published:** 2020-02-12

**Authors:** Matthew McCord, Alicia Steffens, Rodrigo Javier, Kwok-Ling Kam, Kathleen McCortney, Craig Horbinski

**Affiliations:** 1 Department of Pathology, Northwestern University, Chicago, IL 60630 USA; 2 Department of Neurosurrgery, Northwestern University, Chicago, IL 60630 USA

**Keywords:** Glioma, Hypermutator, MGMT, IDH1, Temozolomide, Mismatch repair

## Abstract

A subset of gliomas has DNA repair defects that lead to hypermutated genomes. While such tumors are resistant to alkylating chemotherapies, they may also express more mutant neoantigens on their cell surfaces, and thus be more responsive to immunotherapies. A fast, inexpensive method of screening for hypermutated gliomas would therefore be of great clinical value. Since immunohistochemistry (IHC) for the DNA mismatch repair (MMR) proteins Msh2, Msh6, Mlh1, and Pms2 is already used to screen for hypermutated colorectal cancers, we sought to determine whether that panel might have similar utility in gliomas. MMR IHC was scored in 100 WHO grade I-IV gliomas (from 96 patients) with known tumor mutation burden (TMB), while blinded to TMB data. Cases included 70 grade IV GBMs, 13 grade III astrocytomas, 4 grade II astrocytomas (3 diffuse astrocytomas and 1 pleomorphic xanthoastrocytoma), 1 grade I pilocytic astrocytoma, 2 grade III oligodendrogliomas, 7 grade II oligodendrogliomas, and 3 grade I glioneuronal tumors. Eight of 100 tumors showed loss of one or more MMR proteins by IHC, and all 8 were hypermutated. Among the remaining 92 gliomas with intact MMR IHC, only one was hypermutated; that tumor had an inactivating mutation in another DNA repair gene, *ATM*. Overall accuracy, sensitivity, and specificity for DNA MMR IHC compared to the gold standard of TMB were 99, 89, and 100%, respectively. The strongest correlates with hypermutation were prior TMZ treatment, *MGMT* promoter methylation, and *IDH1* mutation. Among the 8 MMR-deficient hypermutated gliomas, 4 (50%) contained both MMR-lost and MMR-retained tumor cells. Together, these data suggest that MMR IHC could be a viable front-line screening test for gliomas in which immunotherapy is being considered. They also suggest that not all cells in a hypermutated glioma may actually be MMR-deficient, a finding that might need to be considered when treating such tumors with immunotherapies.

## Introduction

Gliomas are the most common tumors to arise from the brain parenchyma in adults [20]. Standard of care is maximal safe surgical resection. Grade III and IV gliomas are typically treated with radiation and temozolomide (TMZ), a DNA alkylating agent [25]. Diffusely infiltrating gliomas nearly always recur and lose sensitivity to adjuvant therapy.

When gliomas are challenged with TMZ, recurrent subclones often emerge with inactivating mutations in genes encoding DNA mismatch repair (MMR) enzymes, most commonly *MSH2*, *MSH6*, *MLH1*, and *PMS2*. Loss of function in these genes leads to failure of MMR mechanisms, which is essential for inducing programmed cell death in tumor cells damage by temozolomide, thus contributing to temozolomide resistance in recurrent tumors [6, 12, 30]. Tumor mutation burden (TMB) is normally ~ 1 mutation per megabase (Mb) of DNA [1], but MMR defects can lead to a high mutation burden, which has previously been defined as TMB > 20 mutations/Mb DNA [11–13]. In previously published work, this has been referred to as a “hypermutator” or “hypermutated” phenotype. Hypermutated tumor cells tend to display more mutant proteins on their surfaces, making them potentially more vulnerable to immunotherapies like PD-1 and PD-L1 checkpoint inhibitors [3, 10]. Other forms of hypermutated cancers have shown promising responses to immune checkpoint inhibitors [21], and there is great interest in this strategy for hypermutated gliomas [5, 11].

Next generation sequencing (NGS) is the current gold standard for detecting DNA MMR defects and quantifying TMB. While NGS is a powerful tool, it involves significant cost and turnaround time, compared to routine histopathologic tissue evaluation. Panels which cover over 500 genes, such as Tempus xT, cost several thousand US dollars, and have turnaround times ranging from 10 days to 3 weeks [8]. Smaller, more focused NGS panels, such as Glioseq [19], are less expensive, but often do not cover enough of the genome to reliably determine TMB.

A standardized quartet of IHC stains (Msh6, Msh2, Mlh1, and Pms2) is currently used to detect loss of normal MMR gene expression in colorectal cancers [23]. Because most pathology laboratories already have this MMR IHC panel for routine use, we sought to determine its utility as a screening test for hypermutated gliomas.

## Materials and methods

### Aims, design, and setting

The specific aims of this study are: 1) Determine the reliability of immunohistochemistry for DNA mismatch repair enzymes as a screening test for hypermutation in gliomas; 2) Determine association of hypermutation with the factors of temozolomide therapy, *MGMT* methylation status, *IDH1* mutation status, tumor histotype, WHO grade, patient age, and patient gender, and to compare these results with previously published data. This study was designed as a blinded case review and comparison of immunohistochemistry against the gold standard of next generation sequencing with TMB. Evaluation of tumor characteristics associated with hypermutation was based on a retrospective case-control model.

### Characteristics of participants

The cohort consisted of 100 World Health Organization (WHO) grade I-IV gliomas from the Northwestern Nervous System Tumor Bank with known TMB and MMR gene mutations, as determined by the commercially available targeted NGS panel, Tempus xT, covering approximately 600 genes. Tumors were taken from 96 patients (two patients had two separate tumor resections, and one patient had three separate resections). Summary information for the cohort is contained in Table 1. Gliomas were diagnosed according to 2016 WHO classification. Tumors originally diagnosed prior to the publication of the 2016 WHO updates were re-labeled and classified according to histologic and molecular features per 2016 guidelines. *MGMT* promoter methylation status was determined by pyrosequencing. Case collection was done under a Northwestern Institutional Review Board-approved protocol.

**Table 1:** Cohort characteristics

Age	mean 49.6 years (range 19–85)
Variable	N
Sex	
Male	45
Female	52
WHO grade	
I	4
II	11
III	15
IV	70
Histotype	
Astrocytic	91
Oligodendroglial	9
IDH1 status	
Wild-type	66
Mutant	34
*MGMT* status	
Methylated	45
Unmethylated	49
Unknown	6
Recurrent	
Yes	38
No	62
TMZ treatment	
Pre	67
Post	33
Hypermutated	
Yes	9
No	91
TOTAL	100

*N* = 100 tumors from 96 patients (two females each had two tumors in this cohort, and one male had three tumors). See Additional file 1: **Table S1** for further information on each tumor. *TMZ* temozolomide

### Description of materials and processes

IHC was performed using 4 different primary antibodies, including Msh2 (Cell Marque G219–1129, 1:700), Msh6 (Cell Marque 44 (287 M-15), 1:100), Mlh1 (Leica NCL-L-MLH1, 1:100), and Pms2 (Cell Marque MRQ-28 (288 M-15), 1:50). Formalin-fixed, paraffin-embedded 4 μm thick tissue sections were baked at 60 °C for 30–60 min before being deparaffinized and re-hydrated. Antigen retrieval for Msh6, Msh2, and Pms2 was achieved using a Universal Retrieval (Abcam) buffer in a decloaking chamber reaching 110 °C for 5–20 min. Antigen retrieval for Mlh1 used a citrate buffer (pH 6) in a decloaking chamber reaching 110 °C for 10 min. Slides were cooled to room temperature and washed in TBS before neutralizing endogenous peroxidase (Biocare Peroxidase 1). Slides were then treated with a serum-free casein background block (Biocare Background Sniper) before pre-incubation in a 10% goat serum block for 60 min. Primary antibody was added to the slides for overnight incubation at 4 °C. After incubation, slides were washed in 3 5-min washes with TBS-T before incubating in HRP polymer (Biocare MACH 4 Universal HRP Polymer). Reaction products were visualized with DAB (Biocare Betazoid DAB Chromogen Kit). Slides were counterstained with hematoxylin, dehydrated, and mounted with xylene-based mounting media.

Each IHC marker was examined under light microscopy by two independent reviewers (MM and CH) while blinded to NGS data and TMB. Each IHC marker was scored as “retained” or “lost.” In accordance with published data on MMR enzyme immunohistochemistry, cases were scored as “retained” if uniform intact nuclear staining for the protein was observed [23]. Cases were scored as “lost” if nuclear staining was absent in at least some tumor cells that appeared viable and were not near areas of necrosis or thermal artefact. In tumors with lost MMR expression, the pattern (homogeneous versus heterogeneous) was noted. Neoplastic cells within each glioma were identified and differentiated from non-neoplastic cells by variation in size, shape, and density of nuclear chromatin. Nonneoplastic cells were identified by their overall monomorphic appearance and clues within the context of tissue architecture (e.g. small round cells with high nuclear to cytoplasmic ratio were lymphocytes; spindle-shaped cells surrounding lumens with red blood cells were endothelial cells). The non-neoplastic cells served as internal positive controls. Interobserver discrepancies were resolved by reviewing equivocal cases together.

### Statistical analysis

Linear regression and Spearman correlation analyses were performed using GraphPad Prism 8.3.0.

## Results

### Characteristics of tumors and patients in the cohort

The cohort consisted of 100 gliomas, diagnosed in 96 patients (44 male, 52 female) with NGS and TMB data. The cohort included 70 grade IV GBMs, 13 grade III astrocytomas (all anaplastic astrocytomas), 4 grade II astrocytomas (including 3 diffuse astrocytomas and 1 pleomorphic xanthoastrocytoma), 2 grade III oligodendrogliomas; 7 grade II oligodendrogliomas; and 4 grade I gliomas, including 3 grade I gangliogliomas and 1 pilocytic astrocytoma. Nine tumors were hypermutated by NGS.

Of the 100 gliomas, 96 were located in the cortex (53 left-sided and 43 right-sided). There were 41 frontal, 11 parietal, 35 temporal, 4 occipital, 1 fronto-parietal, 2 fronto-temporal, 1 temporo-parietal, and 1 insular. Of the 4 non-cortical gliomas, 2 originated in the corpus callosum, 1 in the 3rd ventricle, and 1 in the brainstem. A total of 66 gliomas were *IDH1* wild type, while 34 were *IDH1* mutant. A total of 94 gliomas had known *MGMT* promoter methylation status (45 methylated, 49 unmethylated). A total of 62 gliomas were primary (prior to any therapy), while 38 were recurrent post-therapy (surgery, chemotherapy, or radiation) (Table 1). Of the 38 recurrent gliomas, 33 were recurrent post-temozolomide, and 5 were recurrent post-surgery, with or without radiation. A total of 67 gliomas were temozolomide-naïve. Available information on temozolomide dosage and cycles is included in Additional file 1: Table S1. Of the 38 recurrent gliomas, 32 were treated with radiation, 6 were treated with CCNU, and 5 were treated with bevacizumab prior to re-resection.

Regarding patients with multiple resections, one patient had three resections, one primary and two recurrent (Additional file 1: Table S1). Only the third resection (second recurrence) showed hypermutation. Another patient had re-resection of a recurrent, non-hypermutated *IDH1*-mutant anaplastic astrocytoma, which recurred again as a hypermutated GBM on the subsequent re-resection. The third patient had two re-resections of two separate recurrences of a GBM; both tumor samples were hypermutated. One patient, a 31 year old female diagnosed with a non-hypermutated *IDH1*-wild type GBM (tumor #81 in Additional file 1: Table S1), underwent germline DNA sequencing and was found to have a *TP53* mutation, diagnostic of Li-Fraumeni syndrome. No other patients had germline DNA sequenced. None had previously been diagnosed with Lynch syndrome.

### DNA mismatch repair enzyme immunohistochemistry is an accurate screening test

**Fig. 1:**
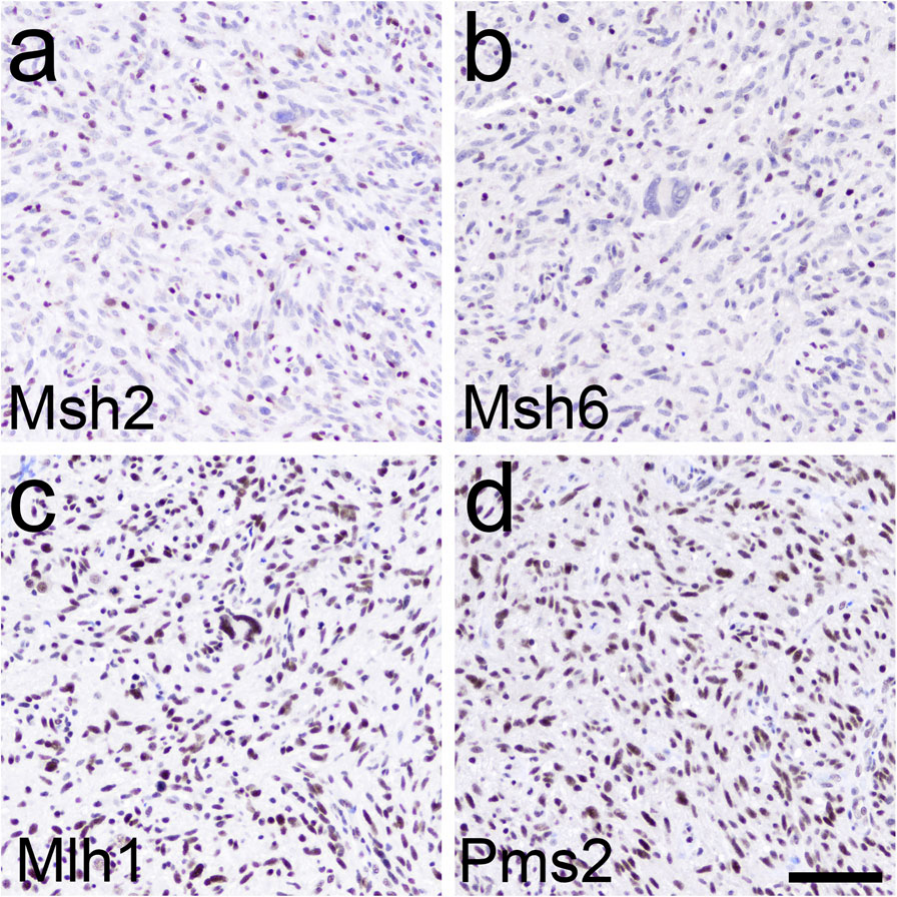
MMR IHC in Tumor 1. The tumor was a recurrent GBM, post-TMZ therapy, in a 57 year-old woman (Table 2). Tumor cells showed loss of Msh2 (**a**) and Msh6 (**b**), and retention of Mlh1 (**c**) and Pms2 (**d**). Note the normal immunostaining within nonneoplastic cells scattered throughout the tumor in (**a**) and (**b**). Scale bar = 100 μm

**Fig. 2:**
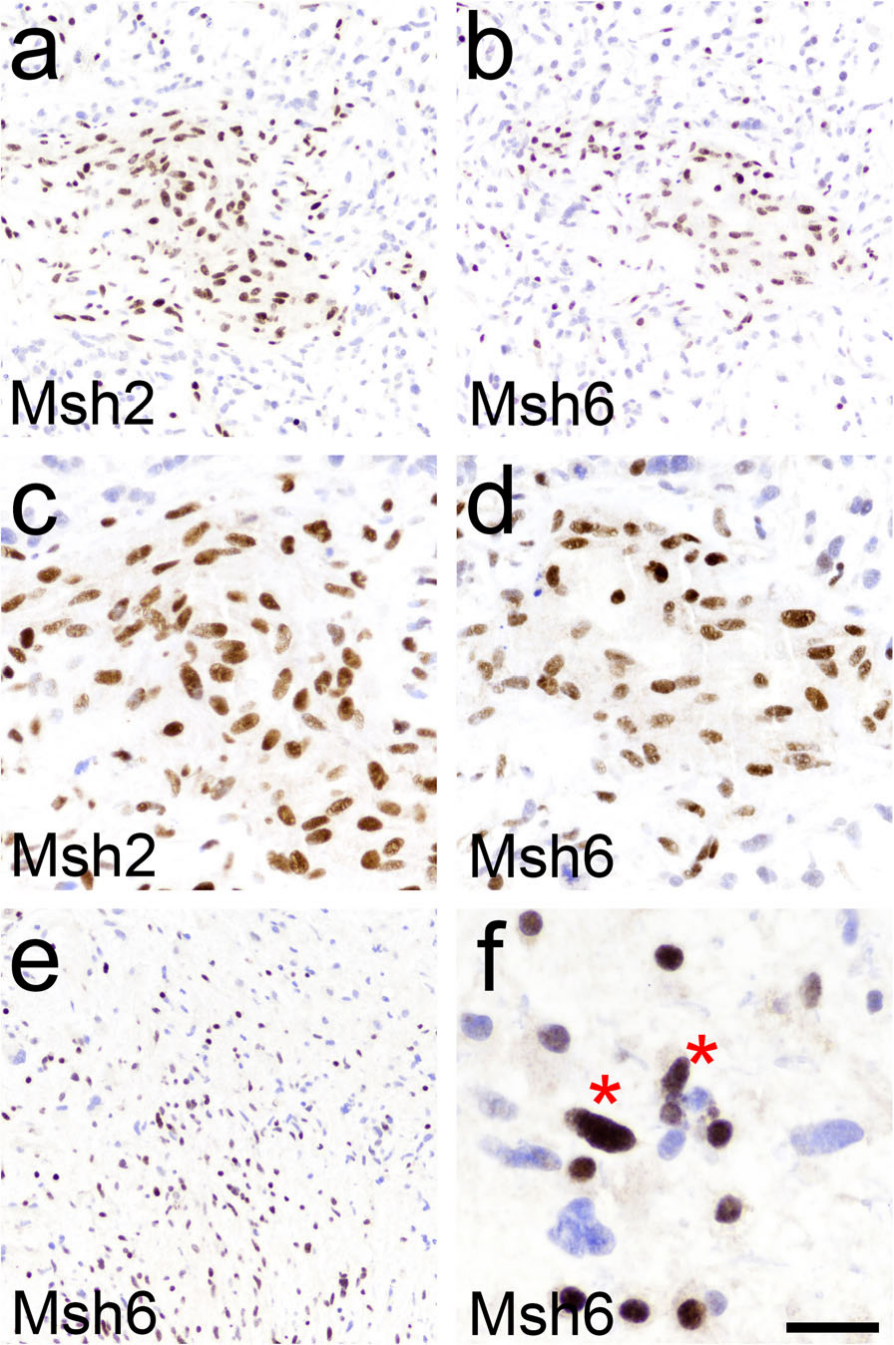
Heterogeneous MMR IHC in hypermutated gliomas. In tumor 7, which was a post-TMZ *IDH1* mutant GBM in a 43 year-old woman (Table 2), clusters of tumor cells retained Msh2 and Msh6 positivity, but were surrounded by Msh2/6-deficient cells (**a**, **b**). Tumor 6 was a post-TMZ *IDH1* wild-type GBM in a 65 year-old woman (Table 2). Msh6 was lost in many glioma cells (**c**), but under high power, it was apparent that a subset of cells with identical tumor nuclear morphology retained Msh6 (**d**, red asterisk). Also note the smaller rounded nuclei in (**d**), which are most likely lymphocytes and/or oligodendrocytes. Scale bar = 100 μm in (**a**, **b**, **e**), 50 μm in (**c**, **d**) and 25 μm in (**f**)

As previously stated, 9 of 100 gliomas were hypermutated by NGS (TMB > 20/Mb). Two of them were separate recurrences from the same patient (Table 2, Additional file 1: Table S1). Eight of 100 gliomas (8%), showed loss of at least one MMR protein by IHC (Fig. 1**,** Table 2). All 8 gliomas with MMR loss were hypermutators, and all 8 had mutations and/or copy number losses in corresponding MMR genes (Table 2). Of the remaining 92 gliomas with intact MMR IHC, only one was hypermutated (TMB = 29.5/Mb). This glioma did not have mutations in *MSH2*, *MSH6*, *MLH1*, or *PMS2*, but instead contained an inactivating splice site mutation in *ATM* (Table 2). Thus, 8 out of 9 hypermutators tested positive on IHC. Sensitivity, specificity, and overall accuracy of MMR IHC for identifying hypermutated gliomas in this cohort was 89, 99, and 98%, respectively. In 4 of 8 gliomas with lost MMR expression, the pattern of loss was clearly heterogeneous, as some tumor cells retained all MMR enzymes, while other cells lost expression of one or more MMR enzymes (Fig. 2).

**Table 2:** Key characteristics of hypermutated gliomas

Tumor #	pre vs post TMZ	IDH1 status	TMB	Msh2	Msh6	Mlh1	Pms2	MMR mutations (AF)	pattern of MMR IHC loss
1	post	WT	> 50.0	lost	lost	retained	retained	*MSH2* stop gain (14.8%) *MSH2* stop gain (9.7%)	homogeneous
2	post	WT	67.8	lost	lost	retained	retained	*MSH2* stop gain (33.0%)	homogeneous
3	post	mut	108.7	retained	lost	retained	retained	*MSH6* stop gain (26.1%) *MSH6* frameshift (16.6%)	homogeneous
4	post	mut	65.0	retained	retained	lost	lost	PMS2 missense (7.2%) *MLH1* copy loss	heterogeneous
5	post	mut	27.0	lost	lost	retained	retained	*MSH2* splice variant (37.8%) *MSH2* stop gain (7.7%)	homogeneous
6	post	WT	58.0	lost	lost	retained	retained	*MSH6* stop gain (27.5%) *MSH6* missense (29.4%)	heterogeneous
7	post	mut	70.5	lost	lost	retained	retained	*MSH2* stop gain (74.0%)	heterogeneous
8	post	mut	20.8	lost	lost	retained	retained	*PMS1* missense (32.5%) *MSH6* missense (11.2%)	heterogeneous
9	post	WT	29.5	retained	retained	retained	retained	*ATM* splice variant (10.5%)	none

Tumor # corresponds to each specimen listed in Additional file 1: **Table S1**. *TMB* tumor mutation burden; *AF* allelic fraction; *IHC* immunohistochemistry

### Variables associated with Hypermutation

**Fig. 3:**
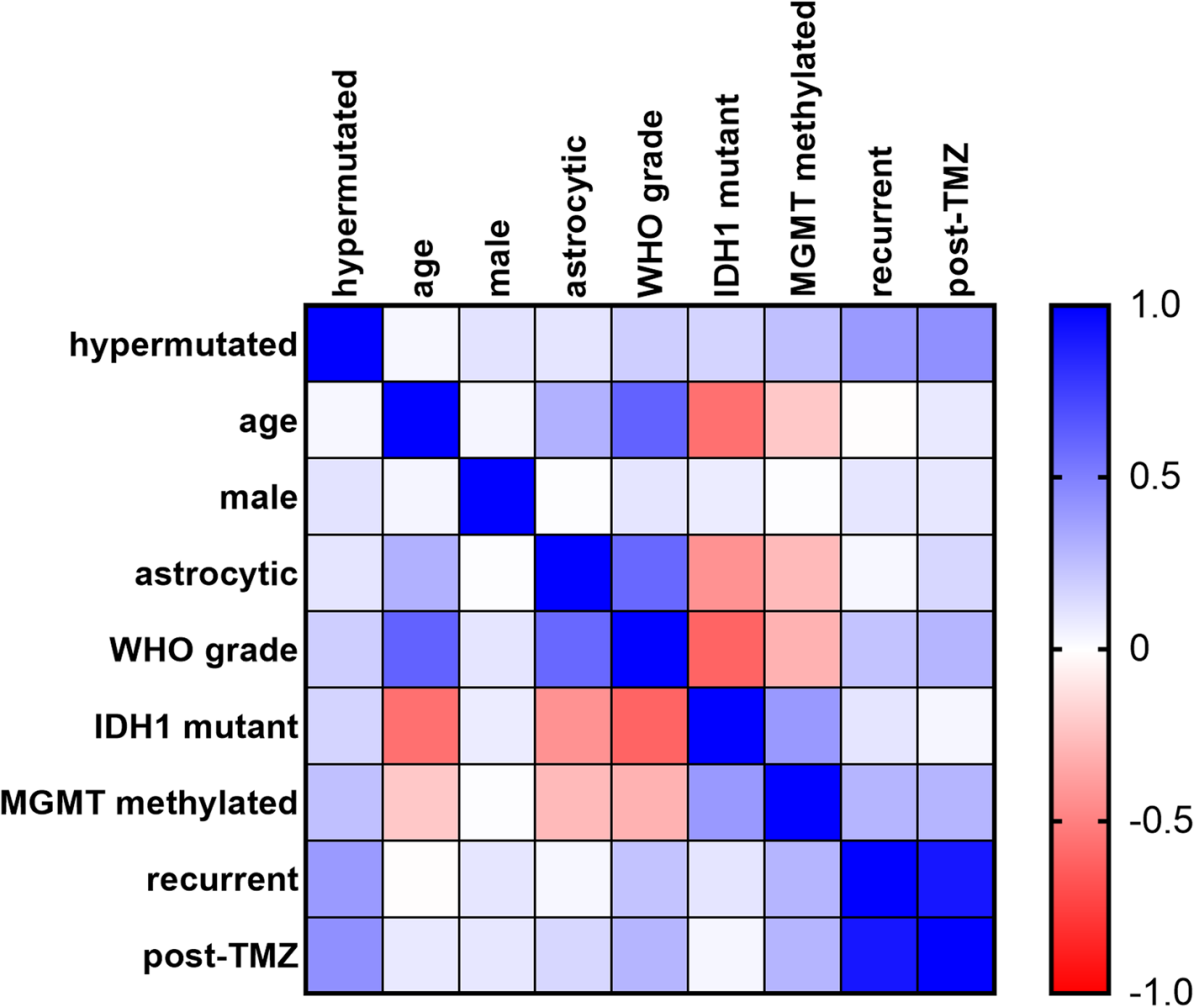
Correlation matrix. Heatmap showing Spearman ρ (rho) correlation coefficients, with 1 = perfect direct correlation, 0 = no correlation, and − 1 = perfect inverse correlation. *N* = 93 tumors (cases without *MGMT* data were excluded, as well as hypermutated tumor #2 since it came from the same patient as tumor #1, see Additional file 1: Tables S1 and S2). TMZ = temozolomide

All 9 hypermutated gliomas had *MGMT* promoter methylation and were post-TMZ, and 5/9 (56%) were *IDH1* mutant (Table 2). Correlation analyses showed that the variables most strongly and significantly associated with hypermutation were prior treatment with TMZ, recurrence, and *MGMT* promoter methylation (Fig. 3, Additional file 1: Table S2). On multivariate regression, which weighs the relative strengths of each variable concurrently with other the variables in determining hypermutation likelihood, *IDH1* mutation was the only variable to reach significance, even though prior TMZ treatment had the highest regression coefficient (Table 3).

**Table 3:** Multiple regression results

variable	regression coefficient	95% CI	t statistic	*P*
**post-TMZ**	0.22	−0.070 to 0.51	1.5	0.13
***IDH1*mutant**	0.15	0.0020 to 0.30	2	0.047
**WHO grade**	0.097	−0.036 to 0.23	1.5	0.15
***MGMT*methylated**	0.072	−0.051 to 0.19	1.2	0.25
**astrocytic**	0.031	−0.22 to 0.29	0.24	0.81
**male**	0.017	−0.091 to 0.13	0.32	0.75
**age**	0.00021	−0.0041 to 0.0045	0.099	0.92
**recurrent**	−0.038	−0.32 to 0.24	0.27	0.79

Variables are listed from largest to smallest regression coefficients, relative to hypermutation. *N* = 93 tumors (cases without *MGMT* data were excluded, as well as hypermutated tumor #2 since it came from the same patient as tumor #1, see Additional file 1: **Tables S1** and S**2**). *TMZ* temozolomide. Multiple *R*^2^ = 0.26, df = 84

## Discussion

Despite their generally aggressive behavior, gliomas tend to have low TMB relative to most other kinds of cancer [1]. However, gliomas that are hypermutated, either at initial presentation or recurrence, may be ideal targets for immunotherapy. Such gliomas usually show increased numbers of infiltrating CD8+ cytotoxic T cells [18, 29], which is consistent with the postulate that hypermutated gliomas are more immunogenic.

Hypermutated gliomas have been a subject of intense investigation for some time, though the reported frequencies of hypermutation vary markedly due to differences in cohort selection. In our screening of over 660 untreated sporadic grade II-IV TCGA gliomas in GlioVis [4], only 15 had detectable mutations in DNA repair enzymes (not shown). But a study by the TCGA consortium showed that 7/19 (36%) TMZ-treated GBMs were hypermutated [26]. Johnson et al. reported hypermutation patterns in 6/10 (60%) post-TMZ tumors, and suggested that most of the acquired mutations were likely directly induced by TMZ [15]. In a separate study of 114 matched pre- and post-treatment GBMs, 17 (15%) showed a hypermutator profile at recurrence; among those 17 cases, 16 had mutations in MMR genes, and showed enrichment for *MGMT* methylation and *IDH1* mutation [28]. Others have verified the association between *IDH1* mutations and hypermutation after TMZ [15, 27, 32]. Among 157 pediatric gliomas, only 9 (6%) were hypermutated, and 7 contained mutations in DNA repair genes [14]. In our own cohort, 9/100 gliomas were hypermutated, all 9 had been previously treated with TMZ, all 9 had *MGMT* promoter methylation, and 5/9 were *IDH1* mutant (Table 2). Screening for hypermutation-associated MMR defects therefore appears to be of greatest value in recurrent, post-TMZ gliomas, especially *MGMT*-methylated and/or *IDH1* mutant tumors (Fig. 3, Table 3, Additional file 1: Table S2).

Although the Msh2, Msh6, Mlh1, and Pms2 IHC panel is used to screen colorectal cancer, mutations in other DNA repair genes have also been reported in post-TMZ hypermutated gliomas, including *MSH4*, *MSH5*, *MLH3*, *PMS1*, *POLE*, and *POLD1* [4, 11, 14, 28]. In our own cohort, we found a hypermutated glioma with an inactivating mutation in yet another gene associated with DNA repair, *ATM* (Table 2) [2]. The MMR IHC panel designed for colorectal cancer detected hypermutated gliomas with good sensitivity and excellent specificity and accuracy. Further studies with a panel using more IHC markers, such as Atm, Msh4, Msh5, or other proteins which may be altered in hypermutated gliomas, could potentially improve the sensitivity further.

Interpretation of MMR IHC in gliomas is relatively straightforward, especially since nonneoplastic cells within the glioma serve as a reliable positive control (Fig. 1). The process is similar to evaluation of ATRX staining in gliomas [7]. MMR staining is lost in areas of necrosis and thermal artifact (not shown), so such regions should not be scored.

Thus far, results from immune checkpoint inhibitors in gliomas have been mixed [5, 16, 22, 31]. While at least partial responses to immune checkpoint inhibitors have been observed in patients whose sporadic gliomas had elevated TMB [9, 17], the best responses have mostly been in glioma patients with an inherited defect in an MMR gene, where 100% of the glioma cells have MMR deficiency [3, 13, 24]. Our data showing frequent heterogeneity of MMR loss in hypermutated gliomas (Fig. 2 and Table 2) underscores the fact that TMB is just a mathematical average of the specimen that was submitted for NGS, and that non-hypermutated subclones of cells may exist in “hypermutated” gliomas [12]. Conversely, hypermutated subclones could potentially exist in tumors whose overall TMB has not yet reached the widely accepted cutoff of 20 mutations/Mb, although we did not see this in our own cohort (not shown).

In sum, DNA MMR enzyme IHC can serve as a rapid, low-cost method of screening for hypermutated gliomas. Highest yield for screening includes recurrent post-TMZ gliomas with *MGMT* promoter methylation and/or *IDH1* mutations. While the current panel used for colorectal cancers has very good sensitivity and excellent specificity, adding more DNA repair IHC markers would further enhance its value. Finally, the observation of heterogeneous loss of staining in some cases is interesting. Future work could elucidate a potential relationship between the heterogeneity of MMR loss of function and degree of tumor response to immune checkpoint inhibitor therapy.

## Conclusions

This study indicates that DNA MMR IHC can serve as a front-line screen for hypermutated gliomas. Focusing on post-TMZ recurrent gliomas that have *MGMT* promoter methylation and/or *IDH1* mutations increases the per-case value of such screening. MMR loss within hypermutated gliomas is often heterogeneous, which could help explain why some hypermutated tumors respond to immunotherapies more than others.

## Supplementary information

**Additional file 1: Table S1.** Detailed clinical and pathologic data for the full cohort of 100 gliomas. Tumors 1–9 are hypermutated and are also shown in **Table S1.** of the main manuscript. The following tumors are from the same patients: 1 and 2; 5, 11, and 12; 7 and 94. Pre-TMZ gliomas were designated as N/A in the TMZ dose and duration columns. Records on temozolomide dosage and cycles were incomplete for many post-TMZ patients, because they had been treated at outside institutions prior to re-resection at Northwestern. “D Astro” = diffuse astrocytoma; “A Astro” = anaplastic astrocytoma; “A Oligo” = anaplastic oligodendroglioma; N/A = not available or not applicable. **Table S2.** Correlation matrix. *N* = 93 tumors (cases without *MGMT* data were excluded, as well as hypermutated tumor #2 since it came from the same patient as tumor #1, see Fig. 2 and Additional file 1: Table S1). Each value represents Spearman ρ (rho) correlation coefficients, with 1 = perfect direct correlation, 0 = no correlation, and − 1 = perfect inverse correlation. *P < 0.05. TMZ = temozolomide.

## Data Availability

Data sharing is not applicable to this article, as no datasets were generated or analyzed during the current study.

## Abbreviations

IDH1: Isocitrate dehydrogenase 1
IHC: Immunohistochemistry
MGMT: O-6-methylguanine-DNA methyltransferase
MMR: Mismatch repair
NGS: Next generation sequencing
TCGA: The Cancer Genome Atlas
TMB: Tumor mutation burden
TMZ: Temozolomide
