# Supplementary material for: The efficacy of DNA mismatch repair enzyme immunohistochemistry as a screening test for hypermutated gliomas
Source: Acta Neuropathol Commun. 2020 Feb 12;8:15. doi: 10.1186/s40478-020-0892-2 (PMC7017562; doi:10.1186/s40478-020-0892-2)
Supplement: Supplementary file 1 — Additional file 1: Table S1. Detailed clinical and pathologic data for the full cohort of 100 gliomas. Tumors 1–9 are hypermutated and are also shown in Table S1. of the main manuscript. The following tumors are from the same patients: 1 and 2; 5, 11, and 12; 7 and 94. Pre-TMZ gliomas were designated as N/A in the TMZ dose and duration columns. Records on temozolomide dosage and cycles were incomplete for many post-TMZ patients, because they had been treated at outside institutions prior to re-resection at Northwestern. “D Astro” = diffuse astrocytoma; “A Astro” = anaplastic astrocytoma; “A Oligo” = anaplastic oligodendroglioma; N/A = not available or not applicable. Table S2. Correlation matrix. N = 93 tumors (cases without MGMT data were excluded, as well as hypermutated tumor #2 since it came from the same patient as tumor #1, see Fig. 2 and Additional file 1: Table S1). Each value represents Spearman ρ (rho) correlation coefficients, with 1 = perfect direct correlation, 0 = no correlation, and − 1 = perfect inverse correlation. *P < 0.05. TMZ = temozolomide. [file 40478_2020_892_MOESM1_ESM.pdf]

Supplementary Data for

The efficacy of DNA mismatch repair enzyme immunohistochemistry as a screening test for hypermutated gliomas

Matthew McCord, MD,[1] Alicia Steffens,[2] Rodrigo Javier,[2] Kwok-Ling Kam, MBBS,[1] Kathleen McCortney, MS,[2] and Craig Horbinski, MD PhD[1, 2]

[1]Department of Pathology, Northwestern University, Chicago, IL 60630, USA

[2]Department of Neurosurgery, Northwestern University, Chicago, IL 60630, USA

Tumor #	Sex	Age	Location	Diagnosis	Histotype	WHO grade	IDH1	MGMT	Primary vs recurrent	Pre vs post-TMZ	TMZ dose	TMZ cycles pre-recurrence	CCNU pre-recurrence?	Bev pre-recurrence?	Radiation pre-recurrence?	Hypermutated?
1	F	56	R Parietal	GBM	Astrocytic	IV	Wild type	Methylated	Recurrent	Post	200 mg/m2	14	N	N	Y	Y
2	F	56	R Temporal	GBM	Astrocytic	IV	Wild type	Methylated	Recurrent	Post	200mg/m2	12	N	N	Y	Y
3	M	53	L Frontal	GBM	Astrocytic	IV	Mutant	Methylated	Recurrent	Post	Incomplete data	Incomplete data	N	N	Y	Y
4	M	63	R Parietal	GBM	Astrocytic	IV	Mutant	Methylated	Recurrent	Post	Incomplete data	10	N	Y	Y	Y
5	M	42	L Temporal	GBM	Astrocytic	IV	Mutant	Unmethylated	Recurrent	Post	Incomplete data	Incomplete data	N	N	Y	Y
6	F	65	L Occipital	GBM	Astrocytic	IV	Wild type	Methylated	Recurrent	Post	200 mg/m2	18	Y	N	Y	Y
7	F	43	L Frontal	GBM	Astrocytic	IV	Mutant	Methylated	Recurrent	Post	150 mg/m2	Non-cyclic	N	N	N	Y
8	M	32	R Frontal	GBM	Astrocytic	IV	Mutant	Methylated	Recurrent	Post	200 mg/m2	6	N	N	Y	Y
9	M	58	R Frontal	GBM	Astrocytic	IV	Wild type	Methylated	Recurrent	Post	Incomplete data	Incomplete data	N	Y	Y	Y
10	F	68	L Occipital	GBM	Astrocytic	IV	Wild type	Methylated	Recurrent	Post	75 mg/m2	Non-cyclic	N	N	Y	N
11	M	39	L Temporal	GBM	Astrocytic	IV	Mutant	Unmethylated	Recurrent	Post	Incomplete data	Incomplete data	N	N	Y	N
12	M	34	L Temporal	A Astro	Astrocytic	III	Mutant	Unmethylated	Primary	Pre	N/A	N/A	N/A	N/A	N/A	N
13	M	40	L Fronto-temporal	A Oligo	Oligodendroglial	III	Mutant	Methylated	Recurrent	Pre	N/A	N/A	N	N	Y	N
14	M	40	L Parietal	GBM	Astrocytic	IV	Mutant	Methylated	Recurrent	Post	200 mg/m2	6	N	N	Y	N
15	M	19	L Frontal	Oligo	Oligodendroglial	II	Mutant	Unmethylated	Primary	Pre	N/A	N/A	N/A	N/A	N/A	N
16	F	24	R Frontal	D Astro	Astrocytic	II	Mutant	Methylated	Primary	Pre	N/A	N/A	N/A	N/A	N/A	N
17	F	69	R Parietal	GBM	Astrocytic	IV	Wild type	Unmethylated	Primary	Pre	N/A	N/A	N/A	N/A	N/A	N
18	F	73	R Parietal	GBM	Astrocytic	IV	Wild type	Methylated	Primary	Pre	N/A	N/A	N/A	N/A	N/A	N
19	F	65	L Frontal	GBM	Astrocytic	IV	Wild type	Unmethylated	Recurrent	Post	Incomplete data	6	N	Y	Y	N
20	F	48	R Parieto-temporal	GBM	Astrocytic	IV	Wild type	Unmethylated	Primary	Pre	N/A	N/A	N/A	N/A	N/A	N
21	M	45	L Temporal	GBM	Astrocytic	IV	Wild type	Unmethylated	Recurrent	Post	150 mg/m2	7	N	N	Y	N
22	M	47	L Temporal	GBM	Astrocytic	IV	Wild type	Methylated	Recurrent	Post	200 mg/m2	12	N	N	Y	N
23	F	73	R Frontal	GBM	Astrocytic	IV	Wild type	Unmethylated	Primary	Pre	N/A	N/A	N/A	N/A	N/A	N
24	M	32	L Frontal	A Astro	Astrocytic	III	Mutant	Methylated	Primary	Pre	N/A	N/A	N/A	N/A	N/A	N
25	F	49	R Frontal	GBM	Astrocytic	IV	Mutant	Unmethylated	Primary	Pre	N/A	N/A	N/A	N/A	N/A	N
26	M	48	R Frontal	A Oligo	Oligodendroglial	III	Mutant	Methylated	Primary	Pre	N/A	N/A	N/A	N/A	N/A	N
27	M	76	R Parietal	GBM	Astrocytic	IV	Wild type	Unmethylated	Primary	Pre	N/A	N/A	N/A	N/A	N/A	N
28	M	24	L Temporal	A Astro	Astrocytic	III	Wild type	Methylated	Primary	Pre	N/A	N/A	N/A	N/A	N/A	N
29	F	67	L Temporal	GBM	Astrocytic	IV	Wild type	Unmethylated	Primary	Pre	N/A	N/A	N/A	N/A	N/A	N
30	M	69	L Frontal	GBM	Astrocytic	IV	Wild type	Methylated	Primary	Pre	N/A	N/A	N/A	N/A	N/A	N
31	F	28	L insular	D Astro	Astrocytic	II	Mutant	Unmethylated	Primary	Pre	N/A	N/A	N/A	N/A	N/A	N
32	M	26	L Frontal	A Astro	Astrocytic	III	Mutant	Unmethylated	Primary	Pre	N/A	N/A	N/A	N/A	N/A	N

33	F	74	L Temporal	GBM	Astrocytic	IV	Wild type	Methylated	Primary	Pre	N/A	N/A	N/A	N/A	N/A	N
34	F	25	L Temporal	GBM	Astrocytic	IV	Wild type	Unmethylated	Recurrent	Pre	N/A	N/A	N	N	N	N
35	F	21	R Frontal	A Astro	Astrocytic	III	Mutant	Methylated	Primary	Pre	N/A	N/A	N/A	N/A	N/A	N
36	M	50	L Frontal	GBM	Astrocytic	IV	Wild type	Unmethylated	Primary	Pre	N/A	N/A	N/A	N/A	N/A	N
37	M	68	R Temporal	GBM	Astrocytic	IV	Mutant	Methylated	Recurrent	Post	Incomplete data	2	N	N	Y	N
38	M	85	R Temporal	GBM	Astrocytic	IV	Wild type	Unmethylated	Recurrent	Post	Incomplete data	3	N	N	Y	N
39	F	53	R Frontal	GBM	Astrocytic	IV	Wild type	Unmethylated	Primary	Pre	N/A	N/A	N/A	N/A	N/A	N
40	M	43	R Temporal	GBM	Astrocytic	IV	Mutant	Unmethylated	Recurrent	Pre	N/A	N/A	Y	N	Y	N
41	M	48	R Temporal	D Astro	Astrocytic	II	Wild type	Unmethylated	Primary	Pre	N/A	N/A	N/A	N/A	N/A	N
42	F	74	L Temporal	GBM	Astrocytic	IV	Wild type	Unmethylated	Primary	Pre	N/A	N/A	N/A	N/A	N/A	N
43	M	63	R Frontal	GBM	Astrocytic	IV	Wild type	Unmethylated	Recurrent	Post	Incomplete data	5 (8 week cycles)	Y	Y	Y	N
44	M	28	R Temporal	A Astro	Astrocytic	III	Mutant	Methylated	Primary	Pre	N/A	N/A	N/A	N/A	N/A	N
45	M	44	R Fronto-temporal	GBM	Astrocytic	IV	Mutant	Methylated	Recurrent	Post	150 mg/m2	6	N	N	Y	N
46	M	48	R Temporal	GBM	Astrocytic	IV	Wild type	Unmethylated	Primary	Pre	N/A	N/A	N/A	N/A	N/A	N
47	F	26	L Frontal	GBM	Astrocytic	IV	Wild type	Unmethylated	Primary	Pre	N/A	N/A	N/A	N/A	N/A	N
48	F	68	R Temporal	GBM	Astrocytic	IV	Wild type	Unmethylated	Primary	Pre	N/A	N/A	N/A	N/A	N/A	N
49	F	82	R Temporal	GBM	Astrocytic	IV	Wild type	Unmethylated	Primary	Pre	N/A	N/A	N/A	N/A	N/A	N
50	F	31	L Parietal	PXA	Astrocytic	II	Wild type	Unmethylated	Primary	Pre	N/A	N/A	N/A	N/A	N/A	N
51	F	48	Corpus callosum	GBM	Astrocytic	IV	Wild type	Methylated	Recurrent	Post	200 mg/m2	14	N	N	Y	N
52	F	48	L Frontal	GBM	Astrocytic	IV	Wild type	Methylated	Recurrent	Post	150 mg/m2	3	N	N	Y	N
53	M	68	R Frontal	GBM	Astrocytic	IV	Wild type	Methylated	Primary	Pre	N/A	N/A	N/A	N/A	N/A	N
54	F	32	R Frontal	A Astro	Astrocytic	III	Mutant	Methylated	Recurrent	Post	150 mg/m2	6	N	N	Y	N
55	F	36	R Parietal	A Astro	Astrocytic	III	Mutant	Methylated	Primary	Pre	N/A	N/A	N/A	N/A	N/A	N
56	F	56	L Frontal	A Astro	Astrocytic	III	Mutant	Methylated	Primary	Pre	N/A	N/A	N/A	N/A	N/A	N
57	F	21	R Frontal	Oligo	Oligodendroglial	II	Mutant	Methylated	Primary	Pre	N/A	N/A	N/A	N/A	N/A	N
58	M	33	3rd Ventricle	Ganglioglioma	Glioneuronal	I	Wild type	N/A	Primary	Pre	N/A	N/A	N/A	N/A	N/A	N
59	F	47	L Temporal	A Astro	Astrocytic	III	Wild type	Methylated	Primary	Pre	N/A	N/A	N/A	N/A	N/A	N
60	F	29	R Parietal	A Astro	Astrocytic	III	Mutant	Unmethylated	Primary	Pre	N/A	N/A	N/A	N/A	N/A	N
61	F	48	R Temporal	GBM	Astrocytic	IV	Wild type	Unmethylated	Primary	Pre	N/A	N/A	N/A	N/A	N/A	N
62	F	76	R Fronto-parietal	GBM	Astrocytic	IV	Wild type	Unmethylated	Primary	Pre	N/A	N/A	N/A	N/A	N/A	N
63	M	74	R Temporal	GBM	Astrocytic	IV	Wild type	Unmethylated	Primary	Pre	N/A	N/A	N/A	N/A	N/A	N
64	F	48	L Frontal	GBM	Astrocytic	IV	Mutant	Methylated	Primary	Pre	N/A	N/A	N/A	N/A	N/A	N
65	F	37	R Frontal	Oligo	Oligodendroglial	II	Mutant	Methylated	Primary	Pre	N/A	N/A	N/A	N/A	N/A	N
66	F	35	L Temporal	GBM	Astrocytic	IV	Wild type	Methylated	Recurrent	Post	150 mg/m2	12	N	N	Y	N

67	M	62	R Temporal	GBM	Astrocytic	IV	Wild type	Unmethylated	Primary	Pre	N/A	N/A	N/A	N/A	N/A	N
68	M	62	Corpus callosum	GBM	Astrocytic	IV	Wild type	Unmethylated	Primary	Pre	N/A	N/A	N/A	N/A	N/A	N
69	F	35	R Temporal	GBM	Astrocytic	IV	Wild type	Unmethylated	Recurrent	Post	150 mg/m2	9	N	N	Y	N
70	M	71	R Frontal	GBM	Astrocytic	IV	Wild type	N/A	Recurrent	Post	Incomplete data	3	Y	Y	Y	N
71	F	48	R Frontal	GBM	Astrocytic	IV	Wild type	Unmethylated	Primary	Pre	N/A	N/A	N/A	N/A	N/A	N
72	F	41	L Frontal	GBM	Astrocytic	IV	Wild type	Methylated	Recurrent	Post	200 mg/m2	4	N	N	Y	N
73	M	56	R Frontal	GBM	Astrocytic	IV	Wild type	Methylated	Primary	Pre	N/A	N/A	N/A	N/A	N/A	N
74	M	74	L Occipital	GBM	Astrocytic	IV	Wild type	Unmethylated	Primary	Pre	N/A	N/A	N/A	N/A	N/A	N
75	M	22	L Frontal	Oligo	Oligodendroglial	II	Mutant	Methylated	Primary	Pre	N/A	N/A	N/A	N/A	N/A	N
76	F	55	L Frontal	GBM	Astrocytic	IV	Wild type	Unmethylated	Recurrent	Post	200 mg/m2	6	Y	N	Y	N
77	F	41	L Frontal	Oligo	Oligodendroglial	II	Mutant	Methylated	Primary	Pre	N/A	N/A	N/A	N/A	N/A	N
78	M	31	Brainstem	Ganglioglioma	Glioneuronal	I	Wild type	N/A	Recurrent	Pre	N/A	N/A	N	N	N	N
79	F	25	L Temporal	Pilocytic Astro	Astrocytic	I	Wild type	N/A	Primary	Pre	N/A	N/A	N/A	N/A	N/A	N
80	F	30	L Frontal	Oligo	Oligodendroglial	II	Mutant	Methylated	Recurrent	Pre	N/A	N/A	N	N	N	N
81	F	31	L Temporal	GBM	Astrocytic	IV	Wild type	Unmethylated	Primary	Pre	N/A	N/A	N/A	N/A	N/A	N
82	F	74	L Occipital	GBM	Astrocytic	IV	Wild type	Unmethylated	Primary	Pre	N/A	N/A	N/A	N/A	N/A	N
83	M	50	R Frontal	GBM	Astrocytic	IV	Wild type	N/A	Primary	Pre	N/A	N/A	N/A	N/A	N/A	N
84	M	63	L Frontal	GBM	Astrocytic	IV	Wild type	Unmethylated	Recurrent	Post	Incomplete data	2	N	N	Y	N
85	M	56	L Temporal	GBM	Astrocytic	IV	Wild type	Methylated	Primary	Pre	N/A	N/A	N/A	N/A	N/A	N
86	M	68	L Temporal	GBM	Astrocytic	IV	Wild type	Methylated	Recurrent	Post	200 mg/m2	21	N	N	Y	N
87	M	63	L Frontal	GBM	Astrocytic	IV	Wild type	Unmethylated	Primary	Pre	N/A	N/A	N/A	N/A	N/A	N
88	F	26	L Frontal	A Astro	Astrocytic	III	Mutant	Methylated	Primary	Pre	N/A	N/A	N/A	N/A	N/A	N
89	F	58	R Frontal	GBM	Astrocytic	IV	Wild type	Unmethylated	Primary	Pre	N/A	N/A	N/A	N/A	N/A	N
90	M	45	L Temporal	GBM	Astrocytic	IV	Wild type	Unmethylated	Primary	Pre	N/A	N/A	N/A	N/A	N/A	N
91	F	76	L Temporal	GBM	Astrocytic	IV	Wild type	Unmethylated	Recurrent	Post	200 mg/m2	6	N	N	N	N
92	F	53	L Frontal	Oligo	Oligodendroglial	II	Mutant	Methylated	Recurrent	Post	75 mg/m2	5	Y	N	N	N
93	F	56	R Temporal	GBM	Astrocytic	IV	Wild type	Unmethylated	Primary	Pre	N/A	N/A	N/A	N/A	N/A	N
94	F	42	L Parietal	A Astro	Astrocytic	III	Mutant	Methylated	Recurrent	Post	Incomplete data	Incomplete data	N	N	Y	N
95	F	59	L Temporal	GBM	Astrocytic	IV	Wild type	Unmethylated	Primary	Pre	N/A	N/A	N/A	N/A	N/A	N
96	F	53	L Parietal	GBM	Astrocytic	IV	Wild type	Unmethylated	Primary	Pre	N/A	N/A	N/A	N/A	N/A	N
97	M	41	R Temporal	GBM	Astrocytic	IV	Wild type	Unmethylated	Primary	Pre	N/A	N/A	N/A	N/A	N/A	N
98	M	54	L Frontal	GBM	Astrocytic	IV	Wild type	Methylated	Recurrent	Post	200 mg/m2	3	N	N	Y	N
99	F	30	L Temporal	Ganglioglioma	Glioneuronal	I	Wild type	N/A	Primary	Pre	N/A	N/A	N/A	N/A	N/A	N
100	M	66	L Frontal	GBM	Astrocytic	IV	Wild type	Unmethylated	Primary	Pre	N/A	N/A	N/A	N/A	N/A	N

Supplementary Table 1: Detailed clinical and pathologic data for the full cohort of 100 gliomas. Tumors 1-9 are hypermutated and are also shown in **Table 1** of the main manuscript. The following tumors are from the same patients: 1 and 2; 5, 11, and 12; 7 and 94. Pre-TMZ gliomas were designated as N/A in the TMZ dose and duration columns. Records on temozolomide dosage and cycles were incomplete for many post-TMZ patients, because they had been treated at outside institutions prior to re-resection at Northwestern. “D Astro”=diffuse astrocytoma; “A Astro”=anaplastic astrocytoma; “A Oligo”=anaplastic oligodendroglioma; N/A=not available or not applicable.

	hypermutant	age	male	astrocytic	WHO grade	*IDH1* mutant	*MGMT* methylated	recurrent	post -TMZ
**hypermutant**	1								
**age**	0.03	1							
**male**	0.11	0.037	1						
**astrocytic**	0.10	0.31	0.0047	1					
**WHO grade**	0.19	0.61*	0.099	0.59*	1				
***IDH1* mutant**	0.17	-0.56*	0.074	-0.43*	-0.61*	1			
***MGMT* methylated**	0.25*	-0.22*	0.0056	-0.27*	-0.30*	0.40*	1		
**recurrent**	0.39*	-0.0083	0.098	0.029	0.24*	0.10	0.29*	1	
**post-TMZ**	0.43*	0.082	0.092	0.15	0.29*	0.032	0.29*	0.91*	1

Supplementary Table 2: Correlation matrix. N=93 tumors (cases without *MGMT* data were excluded, as well as hypermutated tumor #2 since it came from the same patient as tumor #1, see **Figure 2** and **Supplementary Table 1**). Each value represents Spearman ρ (rho) correlation coefficients, with 1=perfect direct correlation, 0=no correlation, and -1=perfect inverse correlation. *$P<0.05$. TMZ=temozolomide.
